# Genomic approaches to understanding pregnancy phenotypes and birth outcomes and their links with long-term maternal and offspring health—the value of distinguishing maternal and fetal genetic effects

**DOI:** 10.3389/fgene.2026.1766185

**Published:** 2026-07-17

**Authors:** Ge Zhang, Rachel M. Freathy, Bo Jacobsson, Deborah A. Lawlor

**Affiliations:** 1 Division of Human Genetics, Cincinnati Children’s Hospital Medical Center, Cincinnati, OH, United States; 2 March of Dimes Prematurity Research Center Ohio Collaborative, Cincinnati, OH, United States; 3 Department of Pediatrics, University of Cincinnati College of Medicine, Cincinnati, OH, United States; 4 Department of Clinical and Biomedical Sciences, Faculty of Health and Life Sciences, University of Exeter, Exeter, United Kingdom; 5 Department of Obstetrics and Gynecology, Sahlgrenska Academy, University of Gothenburg, Gothenburg, Sweden; 6 Department of Obstetrics and Gynecology, Western Healthcare Region, Sahlgrenska University Hospital, Gothenburg, Sweden; 7 Department of Genes and Environment, Norwegian Institute of Public Health, Oslo, Norway; 8 Medical Research Council Integrated Epidemiology Unit at the University of Bristol, Bristol, United Kingdom; 9 Bristol Medical School, University of Bristol, Bristol, United Kingdom

**Keywords:** developmental origins of health and disease, genetic architecture, genome-wide association, maternal and fetal genetic effects, Mendelian randomization, multi-variant analysis, polygenic score (PGS), pregnancy

## Abstract

Pregnancy is a biologically complex period with profound implications for maternal and offspring health. Adverse pregnancy outcomes such as pre-eclampsia, gestational diabetes, preterm birth, and growth restriction contribute substantially to maternal and neonatal morbidity and mortality, while also increasing risks of late-onset disorders in both mothers and children. Understanding the genetic basis of these pregnancy phenotypes and birth outcomes is therefore critical for advancing maternal–child health research. Unlike most human traits, pregnancy phenotypes are shaped by both maternal and fetal genomes, which are correlated but exert distinct influences. This dual genetic contribution presents unique challenges for genetic studies and necessitates specialized statistical approaches. In this review, we highlight recent methodological advances in genomic research designed to disentangle maternal and fetal genetic effects. We first discuss single-variant genome-wide association approaches, including conditional analyses, haplotype-based methods, structural equation modeling, and weighted linear models, which enable partitioning of maternal and fetal contributions. We then examine multi-variant strategies such as polygenic scores and Mendelian randomization, which provide insights into causal relationships, shared genetic architectures, and long-term health consequences. Finally, we explore approaches to characterizing the genetic architecture of pregnancy phenotypes, including methods for partitioning maternal and fetal genetic contributions and genetic correlation analyses with other health-related traits. Together, these genomic approaches have begun to clarify the complex interplay between maternal and fetal genomes in shaping pregnancy outcomes and their links to later-life health in both mothers and offspring. By emphasizing methodological innovation rather than empirical findings, this review provides a framework for future studies aiming to unravel the genetic underpinnings of pregnancy phenotypes and their connections with long-term health.

## 1 Introduction

Pregnancy is a biologically complex period that has lasting effects on the health of both mothers and their children. Pregnancy complications and adverse birth outcomes like pre-eclampsia, gestational diabetes, preterm birth, and small for gestational age contribute significantly to maternal and neonatal morbidity and mortality (Blencowe et al., 2012; Abalos et al., 2013; Finken et al., 2018). These adverse outcomes are also linked to increased risks of chronic diseases later in life for both mother and offspring (Shou et al., 2019; D’Agostin et al., 2023; Ahmed et al., 2024). Understanding the genetics of these phenotypes is therefore essential for advancing our knowledge of human pregnancy and improving maternal–child health worldwide.

In this review, we use the term pregnancy phenotypes to refer broadly to measurable traits or clinical characteristics related to pregnancy. These include maternal physical and physiological traits during pregnancy, pregnancy complications (e.g., pre-eclampsia, gestational diabetes), fetal and birth outcomes (e.g., birth weight, gestational duration), and postpartum conditions (e.g., postpartum hemorrhage, depression). Unlike most common human traits, these phenotypes are uniquely shaped by both maternal and fetal genomes, which are genetically correlated but exert distinct influences (Zhang et al., 2018; Evans and Freathy, 2021; Srivastava et al., 2024). This dual genetic influence presents unique challenges for genetic studies of pregnancy phenotypes, as traditional analyses that examine mothers or offspring separately often fail to disentangle maternal and fetal genetic contributions.

This review focuses on statistical genomic methods specifically designed to separate maternal and fetal genetic effects on pregnancy phenotypes. These approaches either utilize genotype data from mother–child pairs or apply advanced statistical models to jointly account for the effects of both genomes. Rather than summarizing empirical findings, we emphasize methodological advances that enable researchers to dissect these genetic contributions and explore their implications for maternal and offspring health. Similar to other complex traits, pregnancy phenotypes are influenced by numerous genetic variants, each exerting a modest effect (McCarthy et al., 2008; Zhang, 2015; Visscher et al., 2017). To address this complexity, we structure our review around three analytical levels (Zhang et al., 2018; Srivastava et al., 2024). In Section 2, we describe statistical methods used in genome-wide single-variant association studies to identify genetic variants or loci associated with pregnancy phenotypes and the estimation of their maternal and fetal genetic associations. In Section 3, we review multi-variant approaches focusing on the cumulative effects of many genetic variants. These include investigation of causal relationships and shared genetic influences using maternal and fetal genetic effects estimated across multiple variants by polygenic scores analysis or multi-variant Mendelian Randomization. In Section 4, we discuss methods for characterizing the genetic architecture of pregnancy traits utilizing genome-wide variation data, including partitioning of maternal and fetal genetic contributions as well as genetic correlation analysis with other health-related outcomes. Throughout, we emphasize the importance of distinguishing maternal and fetal genetic effects and highlight how these methods contribute to understanding the long-term health implications of pregnancy phenotypes.

This is a narrative review rather than a systematic review. The article was developed based on the authors’ domain expertise and close follow-up of the relevant literature. No formal structured literature search strategy was used in preparing this review. Additionally, generative AI tools (Microsoft Copilot) were used exclusively for language refinement, syntax polishing, and text structuring during manuscript preparation; all scientific contents and structure of this review were generated and verified by the authors.

## 2 Genome-wide single-variant association analyses

Genome-wide association (GWA) studies in humans provide an unbiased way to detect associated genetic variants and biological pathways (McCarthy et al., 2008; Visscher et al., 2017). This is particularly valuable for pregnancy phenotypes, as distinct reproductive strategies across species limit the complete translation of animal study findings to human pregnancy (Zhang et al., 2018). Genomic findings from GWA studies can provide novel mechanistic insights, reveal potential interventional targets and new prediction strategies, with the ultimate aim of reducing adverse pregnancy outcomes and their long-term impacts on maternal and offspring health (Srivastava et al., 2024).

The involvement of both maternal and fetal genomes on pregnancy phenotypes is well recognized; however, large-scale GWA studies of pregnancy phenotypes have generally tested associations in mothers and/or infants separately due to the limited availability of genomic data in mother-child pairs and practical barriers in data analysis. Various methods have been used to distinguish the maternal or the fetal sources of the observed genetic associations.

### 2.1 Traditional approach

When genotype data are available for mother-child pairs, a common approach to disentangle maternal and fetal genetic effects is conditional analysis. This is typically implemented by including both maternal and fetal genotypes at the same variant as covariates in a regression model, allowing estimation of their independent contributions to the phenotype. An alternative is to compare effect estimates from maternal and fetal genetic association analyses. Unlike conditional analysis, comparing effect estimates from maternal and fetal association analyses does not require paired mother-child genotype data because it relies on the expected correlation between maternal and fetal genotypes at the population level (r = 0.5). Under the assumptions of additive effects and no simultaneous maternal and fetal effects, a maternal genetic effect estimated in mothers is expected to induce an approximately half-sized association in fetal samples, and *vice versa*. One can even compare summary results from different maternal and fetal GWA studies. These two methods are straightforward to implement and have been widely used in many large-scale GWA studies of pregnancy outcomes (Freathy et al., 2010; Horikoshi et al., 2013; Horikoshi et al., 2016; Zhang et al., 2017). Statistically, both methods require assumptions of pure maternal or fetal genetic effects and additivity allelic effects.

### 2.2 Haplotype-based method

**FIGURE 1:**
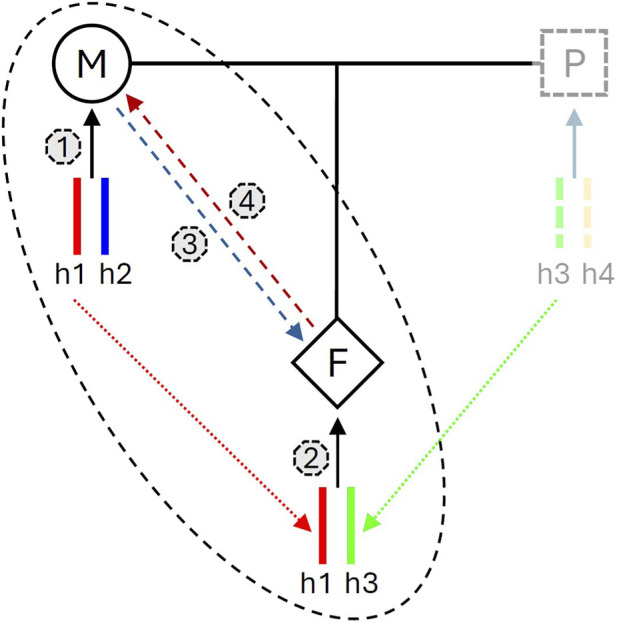
Allele transmission in a duo (or trio) and their possible effects. h1: maternal transmitted alleles can have both maternal (1) and fetal genetic effects (2). h2: maternal non-transmitted alleles only have maternal genetic effect (1) and can be used to proxy maternal causal effect (3, blue dashed arrow); h3: paternal transmitted alleles only have fetal genetic effect (2) and can be used to proxy fetal drive (4, red dashed arrow). The dotted red and green arrows represent allele transmission of h1 and h3. The father (gray square) and the non-transmitted alleles (h4) are shown for completeness but are not typically included in this analysis.

When dense genotype data or genome-wide SNP data are available in mother-child pairs, it is possible to infer haplotype and allele transmission from a mother to her child. In this case, a mother-child pair can be treated as an analytical unit with three alleles: the maternally transmitted allele (h1), the maternally non-transmitted allele (h2), and the paternally transmitted allele (h3) (Figure 1). Therefore, the effects of the three alleles on a pregnancy phenotype (*Y*) can be tested jointly by a single linear model ( $Y=\beta_{1}h1+\beta_{2}h2+\beta_{3}h3+e$ ) (Zhang et al., 2018; Srivastava et al., 2024). This approach explicitly estimates the effects of the three alleles which can influence pregnancy phenotypes differently. For instance, h1 can influence an outcome either through the mother or through the fetus or both; h2 affects through the mother; and h3 acts solely through the fetus. Under certain assumptions, a series of linear hypotheses can be constructed to test for:

- Maternal genetic effect: $\beta_{1}-\beta_{3}+\beta_{2}=0$ assuming no maternal-fetal interaction and no parent-of-origin effect
- Fetal genetic effect: $\beta_{1}-\beta_{2}+\beta_{3}=0$ assuming no maternal-fetal interaction
- Parent-of-origin effect: $\beta_{1}-\beta_{2}=\beta_{3}$ assuming no maternal-fetal interaction

This approach is conceptually attractive and has been used to dissect maternal, fetal genetic effects and parent-of-origin effects in several recent GWA studies for fetal growth measures (Juliusdottir et al., 2021), gestational duration (Solé-Navais et al., 2023), and placenta weight (Beaumont et al., 2023). For example, Beaumont et al. (2023) have demonstrated that the association with placenta weight at the *KCNQ1* locus is conferred by maternally transmitted allele (h1), consistent with the known imprinting pattern at the locus. When paternal genotype data is also available, this approach can also be extended to parent–offspring trios with 4 alleles (h1, h2, h3, and h4—paternally non-transmitted allele) (Kong et al., 2018; Juliusdottir et al., 2021; Beaumont et al., 2023). However, the requirement for complete genotype data in mother-child pairs limits its broader utilization for GWA discoveries.

### 2.3 SEM and WLM

**FIGURE 2:**
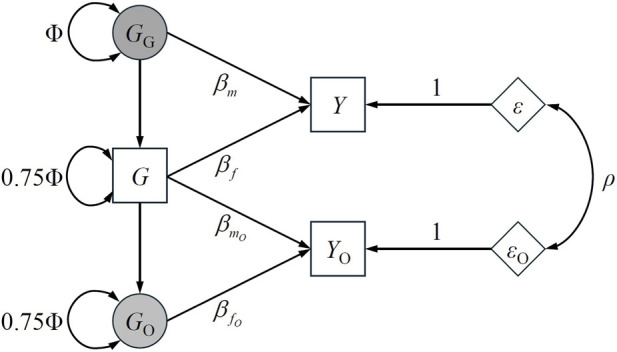
Path diagram of the SEM for fetal and maternal effects on a pregnancy outcome. The three observed variables represented in squares are the mother’s genotype (*G*), her own pregnancy outcome (*Y*) and the outcome in her offspring (*Y*_O_). The latent variables (in circles) are the genotypes for the individual’s mother (*G*_G_) and the genotype of the individual’s offspring (*G*_O_). The total variance of the latent and observed genotypes is set to Φ, such that, var(*G*_G_) = Φ, var(*G*) = 0.75Φ + 0.25Φ, and var (*G*_O_) = 0.75Φ + 0.25Φ. *β*_*m*_ and *β*_*f*_ are path coefficients for maternal and fetal effects, assumed to be the same across generations. *ε* and *ε*_O_ are residual errors for the pregnancy outcome of the individual and their offspring, and ρ is the covariance between them. (Modified from Warrington et al).

A more generalized statistical method for disentangling maternal and fetal genetic contributions is structural equation modeling (SEM) proposed by Warrington and colleagues (Warrington et al., 2018). By jointly modeling an individual’s maternal and fetal genetic effects as well as the latent genetic effects in her mother and offspring due to shared alleles, this method offers a unified framework for estimating maternal and fetal contributions in individuals with birth outcome data of their own and/or their offspring (or more generally, any pregnancy phenotype across generations) (Figure 2). Parameters of this model can be estimated by maximum likelihood using statistical tools like OpenMx (Boker et al., 2011). A major advantage of this approach is its ability to handle various data sets, either mother or infant data, or complete data in mother-child pairs (Evans et al., 2019). This method is computationally heavy and could be challenging when applying to genome-wide studies with large numbers of samples. Additionally, this method requires assumptions of normality of the phenotype and additive maternal and fetal genetic effects (Warrington et al., 2018).

To facilitate the genome-wide application of adjusted maternal and fetal effect estimates, the same authors introduced a weighted linear model (WLM), which calculates unbiased estimates of maternal and fetal effects as a linear combination of effect sizes estimated from maternal and fetal genetic association studies (Warrington et al., 2019). The correlation introduced by sample overlap can be estimated using bivariate LD score regression (Bulik-Sullivan et al., 2015) and subsequently accounted for. Recent GWA studies (Liu et al., 2019; Beaumont et al., 2023) utilized this method to partition maternal and fetal genetic effects using summary results. A recent GWA of placental weight (Beaumont et al., 2023) has extended this method to include paternal data to estimate both parental effects and fetal effects.

### 2.4 Joint analysis of maternal and fetal GWAS

Based on the fact that the maternal and offspring genotypes are correlated, a GWAS study using fetal samples can contribute information to the detection of a maternal effect or *vice versa*. Hwang et al. (2024) have introduced two statistical strategies to improve the power to identify genetic loci with either or both maternal and fetal genetic effects on a pregnancy outcome by jointly analyzing summary results from maternal or fetal GWAS studies. The first one, named as the two-degree-of-freedom test, jointly tests the WLM estimated maternal and fetal effects using a chi-squared test with two degrees of freedom. The second one, named as the one-degree-of-freedom test, combines maternal and fetal GWAS by using weighted meta-analysis assuming exclusive maternal or fetal genetic effects, i.e., doubling the estimated effect from maternal GWA when assuming true fetal genetic effect or *vice versa*. Using simulated empirical data, they showed improved power of these methods over traditional GWA tests in mothers and infants separately. These methods require the same assumptions as the SEM and WLM approaches. However, if the assumption of exclusive maternal or fetal effects does not hold or is incorrectly specified, the one-degree-of-freedom method will yield biased estimates, although it may not lead to an increase in false positive discoveries.

## 3 Methods for multi-variant analyses

**FIGURE 3:**
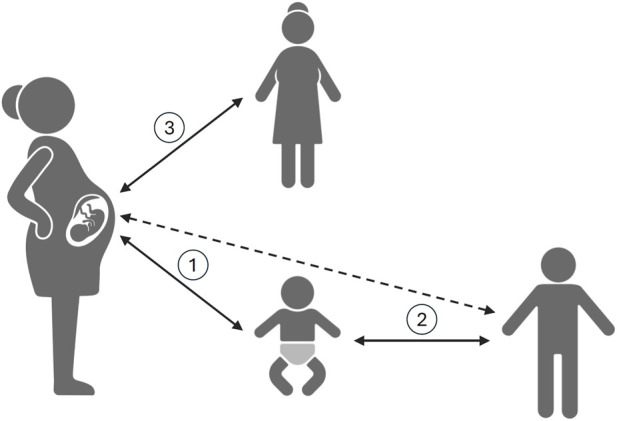
Various associations of pregnancy phenotypes with maternal and offspring health outcomes. 1. Association between maternal phenotype (or exposure) and offspring birth traits. 2. Association between offspring birth traits and offspring later-life health outcomes, which may reflect associations between pregnancy phenotype/exposure and offspring later-life health (dashed link). 3. Association between pregnancy phenotype/exposure and later health outcomes in mothers.

Similar to other common complex traits, pregnancy outcomes are influenced by many genetic variants, each with small effects (McCarthy et al., 2008; Srivastava et al., 2024). Many methods have been developed to investigate the cumulative effects of hundreds to thousands of genetic variants. A widely used approach is to construct polygenic scores (PGS), which aggregate the effects of multiple genetic variants by summing allele counts weighted by their estimated effect sizes (Kullo et al., 2022). While PGS have the potential for predicting genetic risks of complex diseases (Torkamani et al., 2018), their predictive utility for most pregnancy outcomes remains limited, largely due to the small percentage of variance explained by currently identified variants (Honigberg et al., 2023; Srivastava et al., 2024). Despite this, using PGS or a multi-variant approach in Mendelian Randomization (MR) (Davey Smith and Ebrahim, 2003) can improve statistical power and help assess and reduce bias from horizontal pleiotropy (Burgess and Thompson, 2015). Therefore, the section focuses on approaches aimed at uncovering causal relationships and shared genetic architectures underlying associations centered around pregnancy (Figure 3).

Disentangling the various causal effects and the maternal and fetal genetic underpinnings of these associations will enhance our understanding of the etiological mechanisms of pregnancy complications, adverse birth outcomes, and their links to later-life diseases. Addressing these questions requires careful selection of genetic instruments and robust methods for accurately distinguishing and unbiasedly estimating maternal and fetal genetic effects. Many of these studies involve analyses of multiple genetic variants.

### 3.1 Associations between maternal phenotypes or exposures and pregnancy outcomes

**FIGURE 4:**
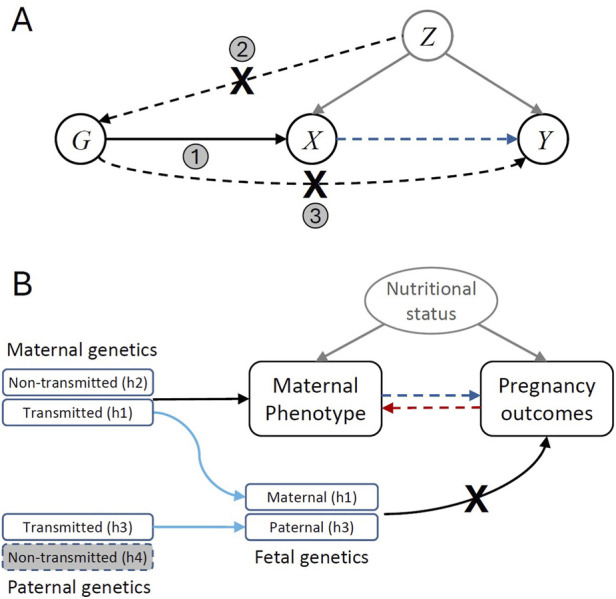
MR and its utilization in determining causal effects of maternal phenotype/exposures on pregnancy outcomes. **(A)** General diagram of MR: Using genetic instrument (*G*) to infer a causal effect (dashed blue arrow) of an exposure (*X*) on an outcome (*Y*). The validity of MR requires the following assumptions to be met: 1) *G* is associated with *X* with sufficient strength (relevance); 2) *G* is independent of any confounding factor *Z* (independence/exchangeability) and 3) *G* affects *Y* only through *X*, not via any other pathway (exclusion restriction). **(B)** Using maternal genotype as genetic instrument to estimate possible causal effect of a maternal phenotype on a pregnancy outcome (blue dashed arrow). The possible bias introduced by the sharing of maternal transmitted alleles (h1) needs to be accounted for or blocked. The red dashed arrow represents possible “fetal drive” from fetus on a maternal phenotype, which can be proxied by paternal transmitted alleles (h3) or fetal genotype with the shared h1 alleles having been adjusted for.

The association between a maternal phenotype or exposure and a pregnancy outcome can arise from 1) maternal causal effects, 2) shared environmental factors and 3) shared genetics due to the transmitted alleles. Mendelian randomization (MR), which uses genetic variants as instrumental variables to infer causal effect (Davey Smith and Ebrahim, 2003), has been widely used to test the causal effect of maternal exposure on birth outcomes (Lawlor et al., 2017) (Figure 4).

Most maternal phenotypes or exposures of interest (*X*) are influenced by many genetic variants with small or moderate effects. To improve the instrumental strength, a common practice is to choose multiple variants associated with *X* as the genetic instrument (*G*) (Burgess et al., 2013; Burgess et al., 2016; Dudbridge, 2021). The assumption of independence between *G* and environmental confounders *Z*—also referred to as exchangeability in causal inference literature (Corbin et al., 2025)—is generally considered valid in MR analysis because alleles are randomly assigned at conception and should be independent of lifestyle or socioeconomic confounders. However, it is difficult to ensure “exclusion restriction” because of possible horizontal pleiotropy, i.e., some of the selected genetic variants might directly, or in linkage disequilibrium (LD) with other variants, influence *Y* through biological pathways other than *X*. The recommendations for variant selection to avoid possible horizontal pleiotropy and the statistical methods to detect such problems and reduce the bias have been reviewed extensively (Evans and Davey Smith, 2015; Sanderson et al., 2022; Burgess et al., 2023).

When utilizing MR to study pregnancy outcomes, ensuring the exclusion restriction can be further complicated by the transmission of maternal alleles (h1 allele in Figure 1), which may affect the pregnancy outcomes through fetal genetic effects (Lawlor et al., 2017; Pingault et al., 2018). To resolve this issue, multiple strategies including the conditional analysis (Tyrrell et al., 2016), using maternal non-transmitted alleles (h2) (Zhang et al., 2015) or SEM/WLM (Evans et al., 2019) have been employed to exclude fetal genetic effects when inferring maternal causal effects.

For example, Tyrrell et al. (2016) studied the causal effects of maternal obesity-related traits on birth weight using maternal genetic scores adjusted for fetal genotypes. This method requires complete data in mother–child pairs and assumptions of additivity of allelic effects and no maternal/fetal interactions. When genetic variants can also influence exposure or the outcome through a paternal genetic effect, or have a parent-of-origin effect, the conditional method may also suffer from collider bias (Lawlor et al., 2017).

The haplotype-based approach is robust to violation of allelic additivity and collider bias; however, it also requires data in mother-child pairs and has reduced power due to the exclusion of the instrumental information carried by the maternal transmitted alleles (h1) (Zhang et al., 2015; Pingault et al., 2018). To improve the power, Chen et al. (2020) estimated maternal effect from all three alleles in mother-child pairs; however, it requires the assumption of additivity of allelic effects. This approach has been applied in a recent study (Huang et al., 2024) to explore the maternal causal effects of maternal traits and fetal genetic effects on birth outcomes–broadly referred to as “intergenerational MR.”

The requirement for mother-child pair data limits broader application of these two methods because of the paucity of such data sets. In contrast, SEM or the WLM methods enable the estimation of maternal or fetal genetic effects using large biobanks containing data from many individuals with information on their own birth outcomes and/or their offspring, or from summary results generated by different large maternal and fetal GWA studies. These benefits facilitate estimation of the causal effects of various maternal phenotypes or exposures on offspring outcomes via large-scale two-sample MR (Lawlor, 2016).

As shown in Figure 4B, it is also possible that the fetus can influence maternal pregnancy phenotypes—a phenomenon referred to as “fetal drive” (Chen et al., 2020). This occurs through fetal genotype-dependent effects on the placenta and fetus, which can modulate maternal physiology through mechanisms such as endocrine signaling, trophoblast invasion, and fetal growth. In these analyses, instead of focusing on the maternal genetic effect, the fetal effect will be used as the causal anchor. For example, MR studies have utilized paternally transmitted alleles (h3) (Chen et al., 2020) or fetal genetic associations estimated by WLM (Beaumont et al., 2023) to proxy fetal effects. These analyses have provided evidence for causal influences of fetal growth on maternal blood pressure and of placental weight on the risk of preeclampsia.

### 3.2 Life-course association of pregnancy outcomes in offspring

Many pregnancy outcomes are associated with various later-life diseases in offspring. Elucidation of the underlying causal mechanisms may provide insights into early prevention and intervention strategies. Two competing but not mutually exclusive hypotheses have been proposed to explain these associations, i.e., the fetal origin hypothesis and the fetal insulin hypothesis. The fetal origins hypothesis (Barker, 1990) emphasizes the negative impact of adverse *in utero* exposures on fetal development and in turn causally influences the future risk of cardiometabolic diseases in adulthood. The fetal insulin hypothesis (Hattersley and Tooke, 1999), on the other hand, emphasized the role of shared genetic factors. For example, genetic variants that affect insulin secretion may contribute to both lower birth weight and increased susceptibility to type 2 diabetes later in life in the offspring.

The above discussed methods for causal inference between maternal phenotypes/exposures on pregnancy outcomes can be readily used to investigate the long-term causal impacts of adverse pregnancy exposures or pregnancy outcomes on late onset disorders. Similarly, to avoid the bias caused by the fetal alleles associated with both the exposure and the outcomes, estimation of the causal effects needs to be based on maternal genetic effects. These studies usually require information on late-onset disorders or cardiovascular risk factors in offspring from birth cohorts followed up to adulthood or biobank data linked to Electronic Medical Records (EMR). For example, Moen et al. (2020) investigated whether a PGS of maternal SNPs associated with offspring birthweight is also associated with offspring cardiometabolic risk factors, after controlling for offspring PGS. Leinonen et al. (2024) assessed the relationship between intrauterine growth and five common health outcomes using mother-child pair data from FinnGen. Both studies suggest maternally influenced intrauterine growth is not a major determinant of adverse cardiometabolic outcomes in offspring.

The shared genetics underlying pregnancy and birth outcomes and later-life diseases can be examined by genome-wide genetic correlation analysis between the GWA summary results of pregnancy and birth outcomes and adulthood phenotypes (Horikoshi et al., 2016; Warrington et al., 2019) (also see next Section: Genetic architecture of pregnancy phenotypes/Estimating genetic correlation). The proportion of phenotype covariance between pregnancy/birth and adult traits attributable to genome-wide variants can also be estimated by the REML method using tools like BOLT-LMM (Loh et al., 2015) when individual-level genotype data are available (Horikoshi et al., 2016).

**FIGURE 5:**
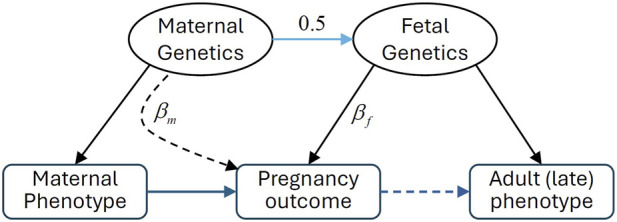
Two possible paths of shared genetic influence between a pregnancy outcome and an adult phenotype. First path: the maternal genetics can influence a pregnancy outcome through a maternal phenotype (indirect maternal effect, path β_m_, dashed line), and it can be associated with an adult phenotype in offspring because of maternal-fetal genetic correlation arising from transmission of maternal alleles (light blue arrow) with the fetal genetics, which influences an adult phenotype. Second path: the fetal genetics can influence both a pregnancy outcome (direct fetal genetic effect, β_f_, solid line) and an adult phenotype. The solid dark blue arrows indicate a direct causal link between the maternal phenotype and a pregnancy outcome, which is required to mediate the indirect maternal effect on the pregnancy outcome. The dashed dark blue arrow suggests a potential, but not required, causal link between a pregnancy outcome and adult phenotype.

It is important to note that there are two possible pathways of shared genetic influences on pregnancy outcomes and adult phenotypes (Figure 5): 1) the transmitted maternal alleles may influence pregnancy outcomes via maternal genetic effects and also affect adult phenotypes in the offspring through fetal genetic effects; 2) the fetal alleles may directly influence both pregnancy outcomes and adult phenotypes in the offspring. To distinguish between these two pathways, it is necessary to disentangle the direct fetal genetic effects from the indirect maternal genetic effects on pregnancy outcomes. Warrington et al. (2019) calculated genetic correlations using LDSC (Bulik-Sullivan et al., 2015) between WLM-adjusted fetal and maternal SNP effect estimates on birth weight and GWAS estimates for various adult traits. They found that the maternal and fetal genetic effects on birth weight can exhibit different genetic correlations with some adult traits. For example, maternal genetic effects were positively correlated with glycemic traits, whereas fetal effects showed negative correlations. Chen et al. (2020) developed a haplotype-based genetic score method to quantify the maternal and fetal contributions of shared genetics to associations between pregnancy outcomes and adult phenotypes. Their findings were consistent with those derived from genetic correlation analyses.

### 3.3 Pregnancy phenotypes and later-life diseases in mothers

The third type of association of medical importance is between pregnancy phenotypes and late-onset disorders in mothers. Intuitively, such associations should be largely explained by shared genetics or environmental factors particularly for closely related pregnancy complications and late-onset disorders, such as between gestational diabetes and type 2 diabetes, gestational hypertension and chronic hypertension. However, as pregnancy is a critical period for mothers, the physiological changes during this period may have long-lasting causal impacts on mothers’ late-life health.

To study the possible causal impact of pregnancy complications, it is important to select genetic variants that are associated solely with the pregnancy complication and not with the late-life outcome to avoid horizontal pleiotropy. However, it is difficult to achieve this given the pregnancy complication and the late-onset disorder under study usually have overlapping genetics. One possible solution is to utilize fetal alleles as genetic instruments similar to the testing of fetal drive on maternal pregnancy phenotypes (Chen et al., 2020). Unlike fetal genetic effects on maternal pregnancy phenotypes, the offspring’s influence on maternal late-life health can extend beyond pregnancy. These may include the lasting effects of fetal drive, as well as lifelong influences through parent-child interactions and broader social or environmental dynamics shaped in part by the offspring. Such extended influence may increase the likelihood of horizontal pleiotropy, as other offspring effects could impact maternal health beyond the pregnancy stage.

Recently Hatton et al. (2025b) introduced a new method – “offspring genotype by proxy” that uses parental genotypes to investigate the causal effects of offspring traits on maternal health outcomes. Although it may have higher bias when there is horizontal pleiotropy through direct paternal effect on the maternal outcome, this method does not need mother-child pair data and can utilize the large numbers of genotyped spousal pairs for causal analyses. The same authors (Hatton et al., 2025a) also developed a MR gene-by-environment interaction approach, that uses parity as a stratification factor to study the effect of pregnancy phenotype on late-life disorders–a true causal effect should be observed only in parous females but not in nulliparous females. However, as there are many differences other than pregnancy between females with or without a live-born child, this method may be subject to bias in causal inference.

## 4 Genetic architecture of pregnancy phenotypes

Understanding the genetic architecture (Mackay, 2001; Lappalainen et al., 2024) of a phenotypic trait involves mapping the total genetic makeup (genotype) to phenotype, determining the proportion of variance explained by genetics (heritability), distribution of allelic effects (Zhang, 2015), and pleiotropy (Watanabe et al., 2019). Many statistical genetic methods have been developed to use genome-wide SNP data to quantify the heritable component of a complex trait or genetic correlation between traits. These techniques have been highly successful in illuminating the genetic basis of numerous human complex traits. However, they are not directly applicable to pregnancy phenotypes due to the joint influence of both maternal and fetal genomes.

### 4.1 GCTA-based methods

One popular method for estimating SNP-heritability using genome-wide data from unrelated individuals is Genome-wide Complex Trait Analysis (GCTA) (Yang et al., 2010; Yang et al., 2011). The basic concept is to estimate the effects of all SNPs as random effects using a linear mixed model. In this framework, the phenotypic variance attributable to genetics can be estimated based on the genetic relatedness matrix (GRM) following: $\text{var}\left(Y\right)=A\sigma_{g}^{2}+I\sigma_{e}^{2}$ . Here, *A* is the GRM estimated from the SNP genotype among individuals, I is identity matrix and the phenotypic variance var(*Y*) is partitioned into random (additive) genetic effects ( $\sigma_{g}^{2}$ ) and residual effects ( $\sigma_{e}^{2}$ ).

**FIGURE 6:**
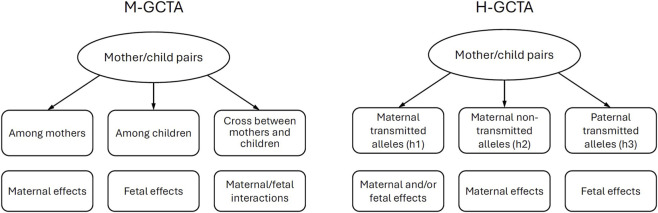
Partitioning of genetic variance in mother-child pairs by M-GCTA (left) and H-GCTA (right). The top row of boxes shows how the genetic relatedness matrices (GRMs) are calculated. The bottom row of boxes shows the source of genetic effects captured by the corresponding GRM. M-GCTA: The “Among mothers” GRM captures maternal genetic effects; the “Among children” GRM captures fetal genetic effects. The “Cross between mothers and children” GRM captures the maternal/fetal interactions, which is the genetic covariance between mother and child. H-GCTA: The GRM for “Maternal transmitted alleles (h1)” captures a combination of both maternal and fetal genetic effects because these alleles are present in both the mother’s and the child’s genome. The GRM for “Maternal non-transmitted alleles (h2)” captures only maternal genetic effects because these alleles are present in the mother’s genome but not transmitted to the child. The GRM for “Paternal transmitted alleles (h3)” captures only fetal genetic effects because these alleles are present only in the child’s genome (assuming no paternal effects).

The first effort to extend GCTA method for resolving maternal and fetal genetic contributions to pregnancy or offspring phenotypes is the M-GCTA (Eaves et al., 2014; Qiao et al., 2020), which uses genome-wide SNP data from mother–child pairs to partition the phenotypic variance in an offspring phenotype into components attributable to the mother’s genome, the child’s genome, the covariance between these two based on three GRMs (Figure 6)—the GRM for all the mothers, the GRM for all the children and the GRM between mothers and children. Another approach is the H-GCTA (Srivastava et al., 2025) which estimates the genetic variance attributable to the three haplotypes: maternal transmitted (h1), maternal non-transmitted (h2) and paternal transmitted (h3) in mother-child pairs based on GRMs calculated from these haplotypes. The results indicated that variance of gestational duration is mainly attributable to h1 and h2, suggesting a stronger maternal genetic contribution. In contrast, variance of fetal size measurements at birth is mainly attributable to h1 and h3, consistent with stronger fetal genetic effects. The utilization of these methods has elucidated the maternal and fetal contributions to many important pregnancy outcomes and both methods can be extended to parent-child trios to detect paternal genetic effects (Eilertsen et al., 2021). These methods have their own limitations. The M-GCTA approach requires assumptions of additive allelic effects, and no parent-of-origin effects. The H-GCTA method is robust to the violation of these assumptions; however, interpretation of the results could be complicated when these assumptions are not met, or the maternal and fetal effects are correlated (Srivastava et al., 2025).

### 4.2 LD score regression

While M-GCTA and H-GCTA offer valuable insights, their reliance on individual genotype data from mother-child pairs limits their wide application. Addressing this, methods leveraging GWAS summary results, such as the approach introduced by Moen et al. (2023), provide a powerful alternative following the genomic structural equation modeling framework (Grotzinger et al., 2019). This method involves two steps: 1) estimating the genetic covariance matrix and its associated sampling covariance matrix from the maternal and fetal GWA summary results; and 2) estimating genetic variance attributable to the maternal genome, the fetal genome, and their covariance (similar to the three components estimated by the M-GCTA method) using SEM. This method has a clear advantage over the GCTA based approaches, as it can use summary results from large GWA studies and does not require individual genotype data from mother-child pairs. In addition, this method can account for covariance due to cryptic relatedness or overlapping samples and can be easily extended to trios.

### 4.3 Estimating genetic correlation

Pregnancy phenotypes are intercorrelated and are associated with many common phenotypes with long-term health implications in both mothers and offspring. Genetic correlation analyses can provide important information on shared molecular pathways and causal relationships between phenotypes (Van Rheenen et al., 2019). The cross-trait LD score regression (LDSC) (Bulik-Sullivan et al., 2015) has been routinely used in many GWA studies of pregnancy outcomes to estimate genetic correlations. However, the conventional LDSC analyses cannot partition the estimated correlation into maternal and offspring origins. To circumvent this limitation, Warrington et al. (2019) performed LDSC analysis using summary results of fetal genetic effects and maternal effects estimated by WLM method, and the authors found positive genetic correlations between many glucose-related traits and maternal effects on birthweight, but negative genetic correlations with fetal effects on birthweight–evidence supporting the fetal insulin hypothesis. This example highlights the importance of distinguishing maternal and fetal genetic effects in genetic correlation analysis of pregnancy phenotypes. The method introduced by Moen et al. (2023) based on genomic SEM (also described in last paragraph) can be applied to multiple traits and provides a more general way to partition the genetic correlation between two traits into maternally mediated and offspring mediated components.

## 5 Discussion and perspectives

**FIGURE 7:**
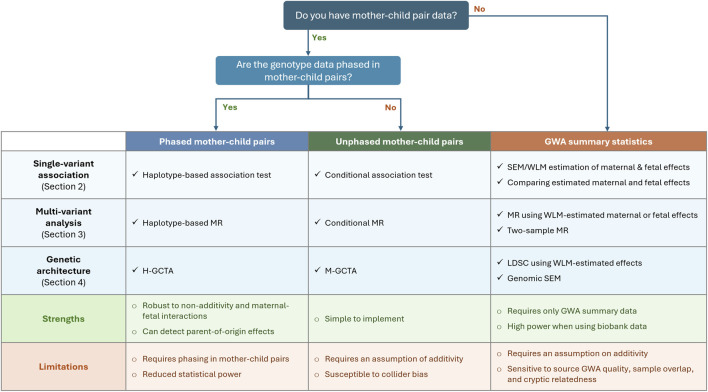
Overview of statistical genomic methods for disentangling maternal and fetal genetic effects on pregnancy phenotypes and birth outcomes. Selection of analytical methods is determined by the availability of mother-child pair genotypes and phasing status. Methods are categorized into three levels: single-variant discovery (Section 2), multi-variant causal inference (Section 3), and global genetic architecture (Section 4). Key strengths and limitations of each approach category are summarized in the bottom rows.

This review has provided an overview of the statistical genomic methods used to study pregnancy outcomes, specifically tailored for the partitioning of maternal and fetal genetic effects. We have covered genetic association tests, multi-variant genetic instrumental analysis, and the estimation of SNP-heritability and genetic correlation using genome-wide variation data. Figure 7 summarizes the major methods discussed in this review, organized by their analytical level and data requirements, along with their key strengths and limitations, to guide the selection of appropriate approaches for future studies.

Emerging resources such as Non-Invasive Prenatal Testing (NIPT) data have demonstrated great potential in genomic research of human pregnancy phenotypes (Liu et al., 2018; Zhen et al., 2024; Liu et al., 2024). However, robust statistical frameworks for applying these data to distinguish maternal vs. fetal genetic effects remain an area for future development (Li et al., 2025). Beyond the joint influence from both maternal and fetal genomes, pregnancy is also characterized by prevalent maternal-fetal cross-talk, dynamic changes, and coordinated regulations. The investigation of these intriguing questions requires future methodology development and innovative applications of existing statistical tools. Looking forward, there are several critical areas where further work is needed. These include more sophisticated methods to study maternal-fetal interactions, and the use of longitudinal approaches to capture the dynamic changes in genetic effects throughout pregnancy. The integration of multi-trait and multi-omics approaches holds promise for revealing core regulatory pathways and bridging the gap between genetic variation and biological function. Pregnancy is a dynamic physiological process driven by maternal adaptation to support placental and fetal development. Multi-omic profiling–integrating longitudinal transcriptomic, proteomic, epigenomic, and microbiome data from maternal, placental, or fetal samples–offers a comprehensive view of these molecular dynamics (Rahnavard et al., 2024). When anchored by genomic data to dissect maternal versus fetal genetic effects, multi-omic integration can map causal pathways and regulatory networks governing pregnancy (Tang et al., 2026). Finally, it is critical to expand research into diverse populations to ensure that genomic discoveries are relevant and beneficial to all.
